# A New Transgenic Mouse Model of Heart Failure and Cardiac Cachexia Raised by Sustained Activation of Met Tyrosine Kinase in the Heart

**DOI:** 10.1155/2016/9549036

**Published:** 2016-05-19

**Authors:** Valentina Sala, Stefano Gatti, Simona Gallo, Enzo Medico, Daniela Cantarella, James Cimino, Antonio Ponzetto, Tiziana Crepaldi

**Affiliations:** ^1^Department of Oncology, University of Turin, 10126 Turin, Italy; ^2^Department of Medical Sciences, University of Turin, 10126 Turin, Italy; ^3^FPO-IRCCS, 10060 Candiolo, Italy; ^4^Department of Molecular Biotechnology and Health Sciences, University of Turin, 10126 Turin, Italy

## Abstract

Among other diseases characterized by the onset of cachexia, congestive heart failure takes a place of relevance, considering the high prevalence of this pathology in most European countries and in the United States, and is undergoing a rapid increase in developing countries. Actually, only few models of cardiac cachexia exist. Difficulties in the recruitment and follow-up of clinical trials implicate that new reproducible and well-characterized animal models are pivotal in developing therapeutic strategies for cachexia. We generated a new model of cardiac cachexia: a transgenic mouse expressing Tpr-Met receptor, the activated form of c-Met receptor of hepatocyte growth factor, specifically in the heart. We showed that the cardiac-specific induction of Tpr-Met raises a cardiac hypertrophic remodelling, which progresses into concentric hypertrophy with concomitant increase in Gdf15 mRNA levels. Hypertrophy progresses to congestive heart failure with preserved ejection fraction, characterized by reduced body weight gain and food intake and skeletal muscle wasting. Prevention trial by suppressing Tpr-Met showed that loss of body weight could be prevented. Skeletal muscle wasting was also associated with altered gene expression profiling. We propose transgenic Tpr-Met mice as a new model of cardiac cachexia, which will constitute a powerful tool to understand such complex pathology and test new drugs/approaches at the preclinical level.

## 1. Introduction

Cachexia has been defined as a “a complex metabolic syndrome associated with underlying illness and characterized by loss of muscle” [1]. Cardiologists have a clear interest in the matter, since 10–15% of chronic heart failure (CHF) patients experience weight loss and wasting of muscle [2], a phenomenon described as cardiac cachexia [3, 4]. The treatment of CHF has made significant advances over the last two decades. Despite this, the clinical perspective remains poor. The situation worsens considerably once muscle wasting is diagnosed [2, 5]. The factors that trigger the progression from clinically and body weight-stable CHF to cardiac cachexia, however, remain poorly understood.

Cachectic patients have significantly reduced fat and bone tissue [6]. However, the major site of protein loss in cachexia is skeletal muscle [7]: skeletal muscle is an important repository of muscle proteins which are mobilized to provide energy substrates and to sustain protein synthesis in other vital organs.

While cancer patients are known to be prone to develop cachexia, cardiac cachexia due to chronic heart failure takes the lead in terms of absolute patient numbers [8]. Despite this, although a large number of animal studies were performed to investigate novel treatments for heart failure, few studies concentrated on cardiac cachexia [3].

Animal models which replicate the clinical findings of cardiac cachexia mainly rely on surgical techniques including myocardial infarction and aortic banding [9]. Two other models of cardiac cachexia have been developed so far [10]. Li et al. demonstrated that cardiac-specific overexpression of calsequestrin, a sarcoplasmic reticulum Ca^2+^ storage protein, resulted in chronic heart failure as evidenced by decreased fractional shortening and cachexia [11]. Kato et al. used the Dahl salt-sensitive rat as a model of cardiac cachexia; these rats showed impaired growth and reduced food intake in comparison with controls [12].

c-Met tyrosine kinase has been identified as the receptor of the hepatocyte growth factor (HGF) [13]. In our previous studies, we aimed at investigating Met's role in the heart, by constitutively activating the HGF/Met system through the expression of Tpr-Met [14]. In the Tpr-Met oncoprotein, the N-terminal region of Tpr, which includes two dimerization motifs, is fused to the tyrosine kinase of Met, which is, thus, constitutively active in the absence of the ligand [15]. By postnatal cardiac-specific expression of Tpr-Met, we generated a model of congestive HF leading to cardiac cachexia.

## 2. Materials and Methods

### 2.1. Ethics Statement

The use of mice for this study and all animal procedures were approved by the Ethical Commission of the University of Turin and by the Italian Ministry of Health.

### 2.2. Conditional Cardiac Tpr-Met Mice

The single transgenics (*α*-MHC-tTA mouse and Tpr-Met-TRE-GFP responder mouse) and bitransgenics were described in [14, 16, 17]. All animals were in FVB 100% background. For genotyping of mouse tail DNA extract, tTa-*α*-MHC and eGFP primers were used. Bitransgenic and control mice were conceived and delivered in the presence of 0.01% Doxycycline Hydrochloride (DOX, MP Biomedicals) in drinking water, in order to maintain suppressing the transgene, during* in utero* development. The day following birth, DOX was removed from drinking water to allow Tpr-Met expression in the postnatal age. All animals were fed standard diet and water* ad libitum* and were maintained on a 12 h light-dark cycle at 23 ± 2°C room temperature. Environmental enrichment was provided.

### 2.3. Echocardiography

Size and function of the left ventricle were evaluated by high-resolution transthoracic m-mode and two-dimensional echocardiography with Vevo 2100 echocardiograph (Visualsonics), as previously described in detail [14, 18]. Fractional shortening and h/r ratio were calculated using standard formulas. Cardiac function was assessed when the heart rate was 350 to 450 bpm.

### 2.4. Sample Collection

Animals were sacrificed by cervical dislocation and organs were immediately rinsed in ice-cold PBS, grossly dried, weighted and immersed in RNAlater (Qiagen) overnight at 4°C, and then deposited at −80°C for long-term storage, to preserve total mRNA/proteins. Hindlimbs were excised and digested overnight with Proteinase K (Euroclone). Tibias were scanned and measured using Image J. The mean of weights of left and right muscles was calculated and used for subsequent statistics. After excision of muscles, tibias were excised and digested with proteases. The mean tibia length was used for normalization.

### 2.5. Western Blot and RT-PCR

Protein and total RNA extracts were prepared and analysed as described [14, 16, 17]. Proteins were separated by 8–10% SDS-PAGE and transferred to Hybond C-Extra membranes (Amersham). Equal protein loading was verified by PonceauS (Euroclone) staining. Membranes were blocked in 10% BSA (Sigma Aldrich), incubated overnight at 4° with the primary antibody diluted in 5% BSA, and probed with horseradish peroxidase- (HRP-) conjugated IgGs. Bands were detected using Supersignal West Pico (Thermo Scientific) by Chemidoc XRS (Biorad). Unsaturated images were used for quantification with Imagelab (Biorad). Gapdh was used for normalization of MF20 protein bands in the same gel [16]; Spectra Multicolor Broad Range or Page Ruler reference protein ladder (Thermo Scientific) was used. All the protein samples compared were loaded on the same gel. *n* = 6 ctrls and *n* = 5 TM for WB.

### 2.6. Histology: Cross-Sectional Area (CSA)

PBS-rinsed muscles were fixed in freshly made 4% paraformaldehyde (Sigma Aldrich) 4–8 hours at room temperature or overnight at 4° and then embedded in paraffin. Transversal 10 *µ*m thick sections of the middle were prepared and stained with hematoxylin and eosin. Images were taken with Leica DMRE microscope. ImageProPlus 5.1 software was used for acquisition. Fiber CSA delimited was measured using Image J as described [14]. At least 250 CSA from nonoverlapping fields were measured. *n* = 7 TM and *n* = 9 ctrls.

### 2.7. Illumina Gene Expression Profiling and Bioinformatical Analysis

Processing of tissue and RNA and Illumina technology were described in [17]. Wild-type and single transgenics were used as controls. Cubic spline-normalized probe intensity data and detection *p* values were obtained using GenomeStudio (Illumina). Subsequent data processing included Log_2_ and Log_2_ Ratio transformation. Expander [19] was used to merge redundant probes by Gene ID and generate the heat map from Log_2_ Ratio values, after standardization (mean 0 and std 1) and using complete linkage type and Pearson correlation similarity measurement. Only genes showing a fold change of more than 1.7 and a *p* value of less than 0.05 were included in the analysis. Expander unsupervised hierarchical clustering was used to identify clusters of up- and downregulated genes.

### 2.8. Statistics

Data are expressed as relative values (mean ± SD). In relative measures, controls are set at 1. Differences between groups were determined by independent two-tailed Student's *t*-test.

## 3. Results and Discussion

### 3.1. Cardiac Concentric Hypertrophy Progresses to Marked Heart Failure with Preserved Ejection Function at P27

To obtain transgenic mice in which expression of Tpr-Met can be specifically induced in cardiac muscle in a regulated manner, we adopted the Tet-Off technology [20]. The Tpr-Met-TRE-GFP responder (Tpr-Met responder) construct was assembled by inserting the cDNAs of Tpr-Met and GFP reporter into the bidirectional plasmid pBI. We thus generated a gain-of-function transgenic model with tetracycline-suppressible expression of Tpr-Met under control of the *α*-myosin heavy chain (*α*-MHC) promoter, for specific expression of the transgene in the heart [14]. Tpr-Met mice were conceived and delivered in the presence of DOX, in order to suppress Tpr-Met expression during* in utero* development. The day following birth, DOX was removed from drinking water to allow permanent Met activation in postnatal cardiomyocytes. We have previously shown that cardiac-specific postnatal expression of Tpr-Met oncoprotein leads to cardiac hypertrophy and Tpr-Met mice die at ~4 weeks after birth (between P25 and P27) with signs of congestive heart failure, lung edema, alopecia, ascites, dyspnea, cyanosis, and lethargy [14].

**Figure 1:**
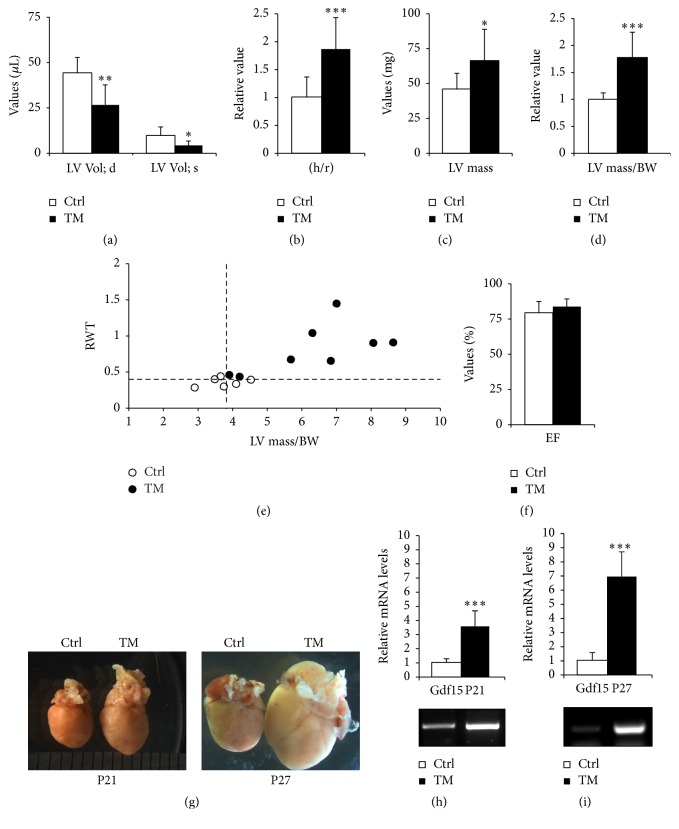
Progressive concentric hypertrophy is accompanied by increased Gdf15 expression in Tpr-Met expressing hearts. (a) At P27, Tpr-Met hearts displayed echocardiographic features of cardiac hypertrophy, including significantly reduced LV Volumes, in both diastole and systole, compared to controls. Hypertrophy was further underlined by a significant increase in thickness/radius ratio (h/r) (b), LV mass (c) and LV mass normalized on body weight (LV mass/BW) (d), and relative wall thickness (RWT) (e) in Tpr-Met mice, compared to controls. (f) At the age of P27, Tpr-Met mice showed preserved ejection fraction (EF). With the increase in the extent of cardiac hypertrophy from P21 to P27, as shown by stereomicroscopy (g), a progressive increase in cardiac Gdf15 mRNA levels was observed from P21 (h) to P27 (i). ^*∗*^ *p* < 0.05; ^*∗∗*^ *p* < 0.01; ^*∗∗∗*^ *p* < 0.005.

At P27, Tpr-Met hearts displayed marked features of cardiac hypertrophy [14], which was also shown by echocardiographic analysis putting in evidence significantly reduced LV Volumes, in both diastole and systole, compared to controls (Figure 1(a)). A pattern fitting in the human classification of concentric hypertrophy [21] was further underlined by the concomitant significant increase in thickness/radius ratio (h/r) (Figure 1(b)), LV mass (Figure 1(c)), and LV mass normalized on body weight (LV mass/BW) in Tpr-Met mice, compared to controls (Figure 1(d)). Plotting relative wall thickness (RWT) and normalized LV mass (Figure 1(e)) showed an important shift of Tpr-Met hearts towards the upper-right corner of the plot, further confirming the diagnosis of concentric hypertrophy. Notably, at the age of P27, Tpr-Met mice did not show systolic dysfunction as assessed by ejection fraction (EF) measurement, but rather a phenotype resembling heart failure with preserved ejection fraction (HFpEF, Figure 1(f)). Indeed, HFpEF is more frequently associated with concentric remodelling and concentric hypertrophy, compared to normal or eccentric geometry [22].

### 3.2. The Progression of Cardiac Hypertrophy to Heart Failure Is Associated with a Progressive Increase in Cardiac Gdf15 Levels

With the massive progressive increase in cardiac hypertrophy from P21 to P27 (Figure 1(g)), also a corresponding dramatic increase in cardiac growth differentiation factor 15 (Gdf15) mRNA levels was observed (Figures 1(h) and 1(i)). Gdf15, also referred to as macrophage-inhibitory cytokine 1 (Mic1), a member of the transforming growth factor-*β* (TGF-*β*) family (Bootcov PNAS 1997), is not expressed in the heart under physiological conditions but increases in response to cardiovascular injuries (i.e., pressure overload, heart failure, and ischemia/reperfusion) and is associated with cardiac remodelling [23]. Higher circulating levels of Gdf15 have been reported in CVDs [24, 25], and such elevations were correlated with disease progression [26]. Notably, Gdf15 has been suggested as a promising diagnostic and prognostic tool for HFpEF [25, 27]. Consistently, we report that, in our model, cardiac levels of Gdf15 increase progressively with the severity of cardiac hypertrophy (Figures 1(g)–1(i)).

Elevated Gdf15 concentrations may be the result of compensating protective mechanisms for tissue repair. Indeed, Gdf15 has shown antihypertrophic and cardioprotective functions [28, 29]. However, beneficial adaptative effects may be detrimental in the long-term. This molecule is overexpressed in cachexia-associated diseases and is capable of modulating appetite [30]. Increased circulating levels of Gdf15 have been reported in patients with several types of cancer [31]. Mice xenografted with tumours overexpressing Gdf15 showed a degree of weight loss proportional to the elevation of serum Gdf15 levels [32]. A direct action of the circulating cytokine on feeding centres in the brain has been also reported [32].

Gdf15 emerges, therefore, as a potential new target for anticachectic therapies. So far, however, no therapies have been identified that decrease the circulating levels of Gdf15 in patients with cardiovascular disease.

### 3.3. Heart Failure Leads to Cardiac Cachexia Syndrome, Characterized by Loss of Body Weight, Reduced Body Weight Gain, and Skeletal Muscle Wasting

Increased heart-to-body weight ratio (cardiac hypertrophy) and enhanced lung-to-body weight ratio (pulmonary congestion) were the most impressive signs of cardiac failure at P27, as we have previously described [14].

**Figure 2:**
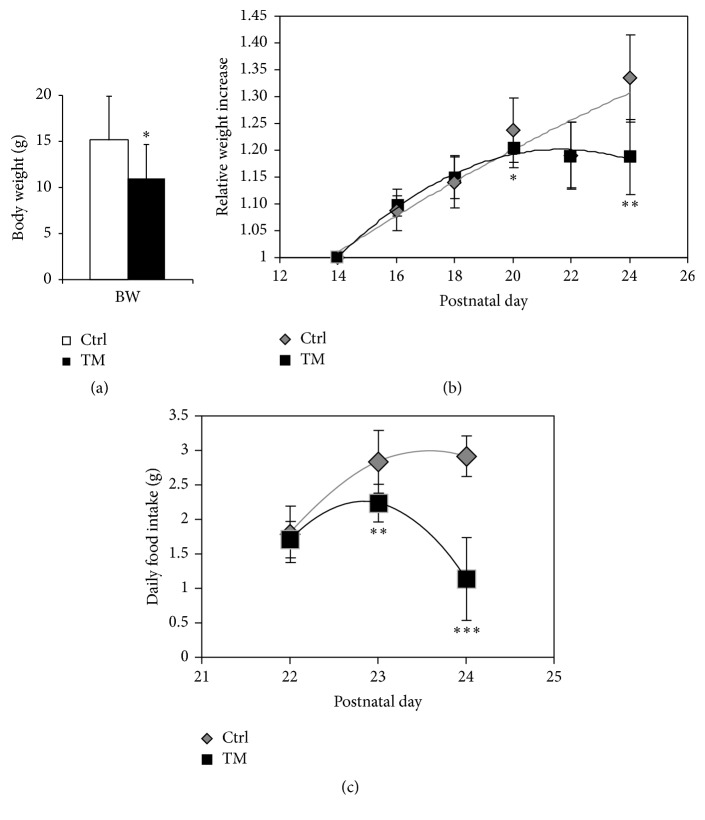
In Tpr-Met mice, progressive concentric hypertrophy leads to reduced body weight and food intake. Tpr-Met mice showed a significant decrease in body weight (BW) (a), body weight gain (b), and daily food intake (c). Tpr-Met mice showed marked decrease in body weight starting from postnatal day 20 (P20) and food intake starting from postnatal day 22 (P22). ^*∗*^ *p* < 0.05; ^*∗∗*^ *p* < 0.01; ^*∗∗∗*^ *p* < 0.005.

To investigate such progression from body-weight stable to failing conditions, each animal was placed in a single case starting from P21. Body weight gain and food intake of Tpr-Met mice and control littermates were evaluated starting from the age of P14 and P22, respectively. At P27, Tpr-Met mice showed marked (>27%) decrease in body weight (Figure 2(a); *n* = 18 controls and *n* = 9 Tpr-Met). According to Anker and Coats, when weight loss higher than 7.5% of the previous normal weight is observed in heart failure patients (for at least six months and without signs of other primary cachectic states), cachexia should be diagnosed [33]. Decreased body weight at P27 resulted from a reduction in body weight gain, which started from postnatal day P20 (Figure 2(b); *n* = 8 controls and *n* = 5 Tpr-Met). Cardiac cachexia involves similar features of other forms of cachexia, including anorexia. Food intake of Tpr-Met mice was still normal at P22 but diminished significantly starting from postnatal day 23 (Figure 2(c); *n* = 8 controls and *n* = 5 Tpr-Met).

**Figure 3:**
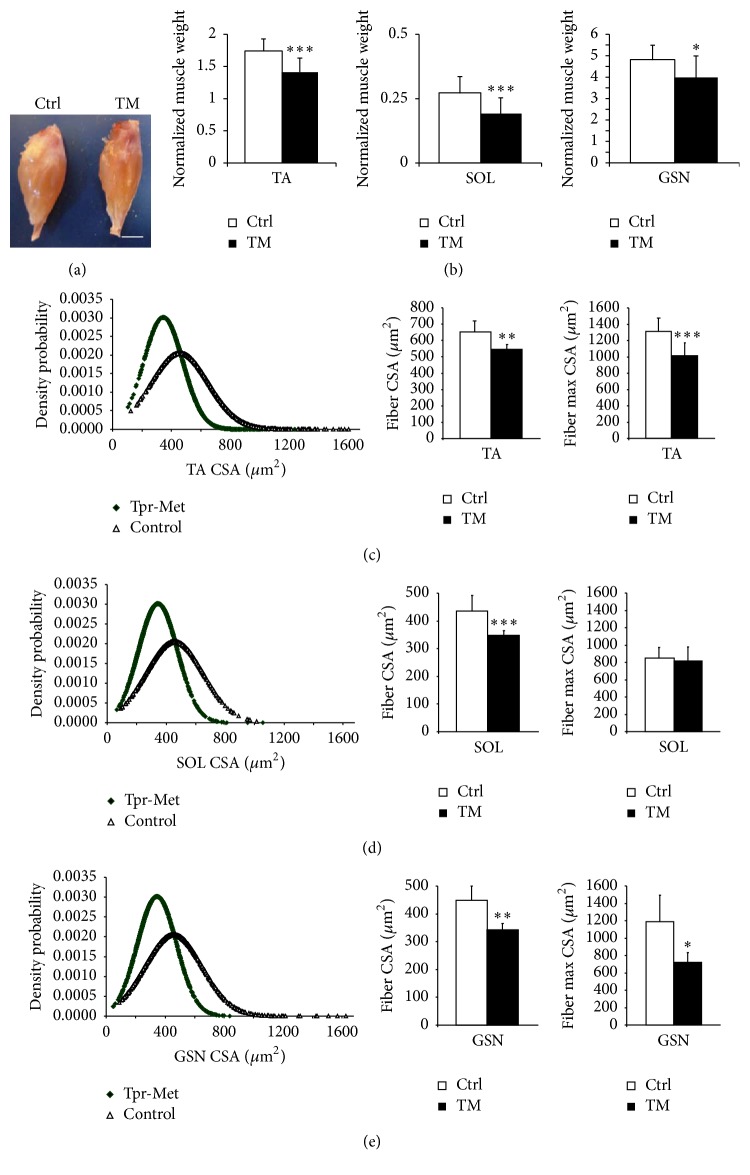
At P27, heart failure in Tpr-Met mice is associated with skeletal muscle wasting. Cachectic Tpr-Met mice had significantly reduced skeletal muscle (a) mass and (b) weight with respect to controls (tibialis anterior: TA, soleus: SOL, and gastrocnemius: GSN). A shift towards smaller fibers was recognized in all three muscles ((c)–(e), left graphs). Accordingly, the mean Cross-Sectional Area (CSA) of TA (c), SOL (d), and GSN (e) muscle fibers of Tpr-Met mice was reduced with respect to controls (middle graphs). The maximum value of CSA was also significantly reduced in TA (c) and GSN (e) muscles (right graphs). Consistently, muscles from Tpr-Met mice showed reduced areas and mass. ^*∗*^ *p* < 0.05; ^*∗∗*^ *p* < 0.01; ^*∗∗∗*^ *p* < 0.005.

According to its definition, cachexia is characterized by loss of muscle. Hence, when signs of congestive heart failure were clear, Tpr-Met mice were sacrificed and tissues were collected and weighed. Muscle weights were normalized to the respective tibia lengths. Cachectic Tpr-Met mice showed wasted hindlimb muscles (Figure 3(a)) and had significantly reduced skeletal muscle weight with respect to controls (TA, SOL, and GSN; *n* = 10 Tpr-Met and *n* = 26 controls; Figure 3(b)). Accordingly, the mean Cross-Sectional Area of TA, SOL, and GSN muscle fibers of Tpr-Met mice was reduced, with respect to controls (*n* = 9 controls and *n* = 7 Tpr-Met for CSA; Figures 3(c)–3(e), left and middle graph). The maximum value of fiber CSA was also significantly reduced in TA and GSN muscles (Figures 3(c)–3(e), right graphs).

In the sum, Tpr-Met mice suffering from heart failure showed a marked decrease in body weight, body weight gain, and food intake and showed skeletal muscle loss due to muscle fiber atrophy, hence recapitulating the phenotype of cardiac cachexia syndrome.

### 3.4. Suppressing Tpr-Met Expression at P21 Rescues Cardiac Hypertrophy and Loss of Muscular Weight

The extent to which hypertrophic remodelling is reversible is poorly known. To this aim, at birth, Doxycycline (DOX) was withdrawn from a group of Tpr-Met mice for 21 days in order to activate Tpr-Met signaling; DOX was subsequently restored to stop Tpr-Met expression. At study completion (P27), a complete rescue of heart hypertrophy and lung edema was obtained when suppressing Tpr-Met expression at P21, since Tpr-Met mice showed normal heart and lung weights [34].

**Figure 4:**
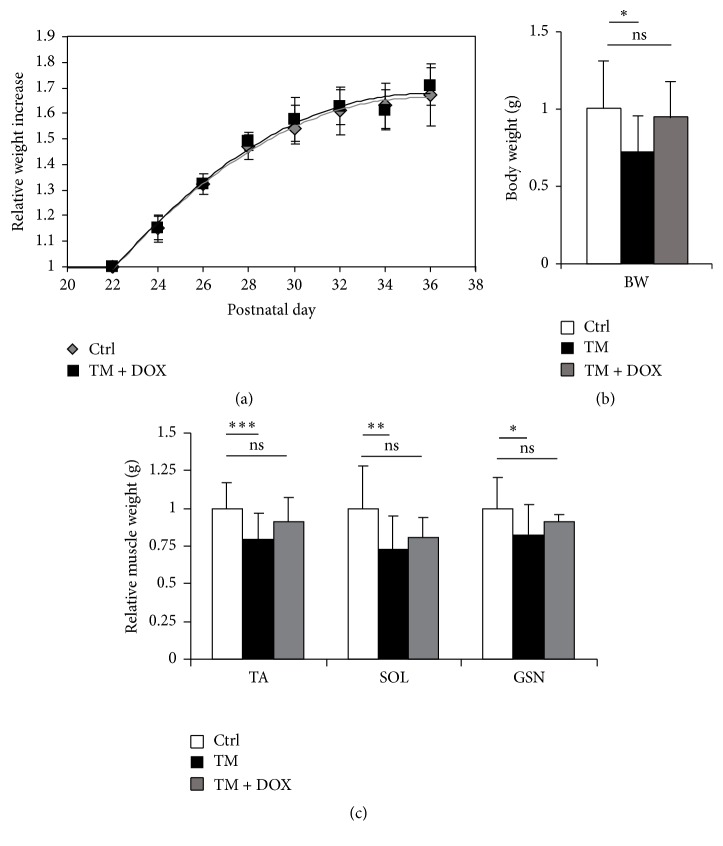
Suppressing Tpr-Met expression at P21 prevents loss of muscular weight at P27. Doxycycline (DOX) was withdrawn from a group of Tpr-Met mice for 21 days in order to activate Tpr-Met signaling; DOX was subsequently restored. The reduction in body weight gain (a) and (b) body weight (BW) was completely rescued when Tpr-Met transgene expression was suppressed by DOX administration (Tpr-Met + DOX) from P21, perfectly overlapping with values from controls for the whole follow-up. (c) When suppressing Tpr-Met expression at P21, the weight of tibialis anterior (TA), soleus (SOL), and gastrocnemius (GSN) muscles was normalized to values overlapping those of controls. ^*∗*^ *p* < 0.05; ^*∗∗*^ *p* < 0.01; ^*∗∗∗*^ *p* < 0.005.

It has been demonstrated that the use of ACE inhibitors and *β*-blockers can potentially delay (and eventually prevent) the onset of cardiac cachexia [3]. Notably, the reduction in body weight gain was completely rescued when Tpr-Met transgene expression was suppressed by DOX administration from P21 (Tpr-Met + DOX P27; *n* = 4), perfectly overlapping with values from littermate controls (*n* = 6) (Figure 4(a)). After such prevention trial, Tpr-Met mice showed normal body weight at P27 (Figure 4(b)), and, consistently, the weight of TA, SOL, and GSN muscles was increased to values comparable with those of controls (*n* = 10 Tpr-Met, *n* = 6 Tpr-Met + DOX P27, and *n* = 26 controls; Figure 4(c)).

These data put in evidence the fact that solving the hypertrophic phenotype, before the appearance of skeletal muscle wasting, can prevent the onset of cardiac cachexia in an effective way, even if cardiac hypertrophy is already manifest.

### 3.5. Gene Expression Profiling of Wasting Gastrocnemius Muscle

**Figure 5:**
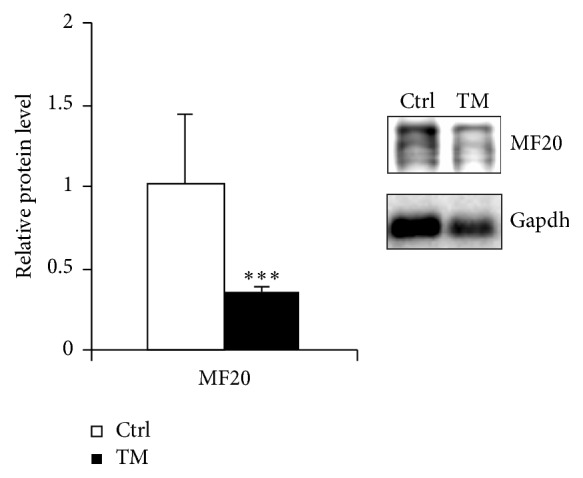
Reduced myosin heavy chain content in gastrocnemius muscle from cachectic Tpr-Met mice. Myosin heavy chain content was quantified in the gastrocnemius muscle (GSN) of cachectic Tpr-Met mice and normalized on Gapdh levels. ^*∗∗∗*^ *p* < 0.005.

For the molecular analysis of wasting skeletal muscle, we concentrated on gastrocnemius (GSN) muscle. In GSN muscle, a marked decrease of MF20 protein level normalized on Gapdh was shown (Figure 5), in accordance with the loss of muscle fiber detected in terms of weight and Cross-Sectional Area.

**Figure 6:**
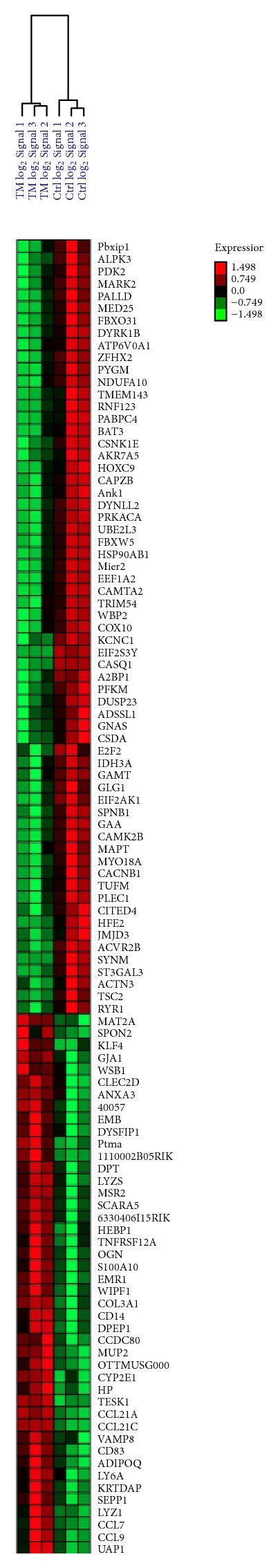
Heat map of the gene expression profiling of wasting gastrocnemius muscle from cachectic Tpr-Met mice. Total RNA from gastrocnemius muscle (GSN) of cachectic Tpr-Met mice was compared to littermate controls. Standardization was applied to Log_2_ absolute intensities. A cut-off of *p* < 0.05 and fold change >1.7 was used. Unsupervised hierarchical clustering was performed and the corresponding heat map was generated. 107 unique genes differentially expressed were identified.

Then, we performed gene expression profiling with Illumina technology on GSN muscle samples. Total RNA from three cachectic Tpr-Met mice was compared to three littermate controls. Standardization was applied to Log_2_ absolute intensities and all subsequent analysis was performed using Expander software. A cut-off of *p* < 0.05 and fold change >1.7 was used. Unsupervised hierarchical clustering was performed and the corresponding heat map was generated (Figure 6). 120 differentially expressed probes were identified and then reduced to 107 unique genes. Among these, 63 were downregulated and 44 genes were upregulated (Supplementary Tables  1 and 2 in Supplementary Material available online at http://dx.doi.org/10.1155/2016/9549036).

A number of genes were downregulated (Supplementary Table  1), including those controlling energy metabolism: muscle glycogen phosphorylase (Pygm) and phosphofructokinase (Pfkm), pyruvate dehydrogenase kinase 2 (Pdk2), NADH dehydrogenase (ubiquinone) 1 alpha subcomplex 10 (Ndufa10), aldo-keto reductase family 7a5 (Akr7a5), and isocitrate dehydrogenase 3 (NAD+) alpha (Idh3a). Also genes regulating protein synthesis were downregulated: eukaryotic translation initiation factor 2, subunit 3 (Eif2s3y), eukaryotic translation elongation factor 1a2 (Eef1a2), and E2F transcription factor 2 (E2f2) as well as myosin XVIIIa (Myo18a) and heat shock protein 90b1 (Hsp90ab1).

We then performed classification of downregulated genes into functional GO categories (Table 1) and KEGG pathways (Table 2). Among the downregulated GO categories, we found translation factor activity, nucleic acid and nucleotide binding, regulation of metabolic process and phosphate metabolic process, muscle system process, generation of precursor metabolites and energy, and cellular protein metabolic and biosynthetic processes. Among the downregulated KEGG pathways, we found tight and gap junctions, galactose metabolism, metabolic pathways, oxidative phosphorylation, and insulin signaling pathway. Altogether, these data suggest an alteration in metabolic activities and growth processes.

On the other hand, among the upregulated genes (Supplementary Table  2), we found tumour necrosis factor receptor superfamily 12a (Tnfrsf12a), also known as the Tweak-receptor (TweakR), as well as a number of cytokines and chemokines. Accordingly with data shown in [35], our work suggests the presence of an inflammatory response in the muscle milieu. Indeed, among the upregulated GO categories and KEGG pathways, there were immune and defense response, chemokine activity, and chemokine signaling pathway cytokine-cytokine receptor interaction.

Finally, several genes regulating ion trafficking, homeostasis, and signaling were either upregulated (S100 calcium binding protein A10 (S100a10), gap junction membrane channel protein alpha 1 (Gja1), cytochrome P450, and family 2e1 (Cyp2e1)) or downregulated (ryanodine receptor 1 (Ryr1), cAMP dependent protein kinase alpha (Prkaca), potassium voltage gated channel, Shaw-related subfamily 1 (Kcnc1), calmodulin binding transcription activator 2 (Camta2), and calcium/calmodulin-dependent protein kinase 2b (Camk2b)). Cation transport GO category and calcium signaling pathway, long-term depression, and long-term potentiation KEGG pathways were enriched in the list of downregulated genes. Indeed, disruptions in calcium signaling have been implicated in cytokine-mediated disruptions in skeletal muscle and function, at least in cancer cachexia [36, 37].

By gene expression profiling and bioinformatical analysis, we found that genes and TFs associated with muscle metabolism, growth, and protein synthesis were negatively modulated. Indeed, the overall net catabolic dominance in heart failure provokes systemic tissue wasting [38], and such downregulation may have contributed to the suppression of growth in wasting muscle.

On the other hand, genes associated with inflammation were increased. Indeed, cytokines may play a significant role in the progression of cardiac cachexia since they may directly affect peripheral skeletal muscle metabolism. A possible underlying pathogenic mechanism for such effect might be found in enhanced activation of the transcription factor NF-*κ*B [39]. In fact, chronic activation of this TF results in skeletal muscle wasting resembling clinical cachexia [40]. Increased expression of proinflammatory cytokines in the skeletal muscle has been demonstrated in cancer [37] and chronic heart failure [35, 41]. Notably, circulating cytokine levels do not correctly reflect tissue levels [35].

## 4. Conclusions

Only a few animal models which replicate the clinical findings of cardiac cachexia have been generated [10]. However, some of these mainly rely on surgical techniques [9]; following these procedures, an increase in resources is required, due to delay in cachexia onset and to the costs due to surgery itself. Moreover, technical issues are still important in limiting the preciseness and reproducibility of heart failure models. This criticism has been overcome by means of transgenic mice and Dahl salt-sensitive rats models. In the present study, we propose a new animal model for research on cardiac cachexia, characterized by high reproducibility and very fast timing.

Some pharmacological treatments for cardiac cachexia have been suggested [42]. Notably, using short-term models of cardiac cachexia could be the best choice for testing the efficacy of a wide range of compounds, from those which have already passed early tests for the development of tolerance to the treatment to new innovative drugs. Notably, in experimental animals, weight loss has been reversed by neutralisation of tumour-produced Gdf15 with a monoclonal antibody [30], encouraging further research to provide a new effective therapy to patients suffering from cardiac cachexia.

## Supplementary Material

Gene expression profiling was performed on GSN muscle samples from cachectic Tpr-Met mice and littermate controls. A cut-off of *p*<0.05 and fold change > 1.7 was used, and 107 differentially expressed genes were identified. Among these, 63 were downregulated and 44 genes were upregulated. The complete gene list is reported in Supplementary Tables 1 and 2.

## Figures and Tables

**Table 1:** Enriched GO molecular function (MF) and biological process (BP) categories in down- and upregulated genes (fold change > 1.7; *p* < 0.05). Only categories significantly associated (*p* value < 0.05) are considered. The name and number of gene ontology (GO) categories, number of enriched genes, raw and corrected *p* values, frequency in the gene set, and the corresponding gene list are reported.

GO category	Number of genes	Raw *p* value	Correct *p* value	Frequency in set (%)	Gene list

Gene set: downregulated genes					
Translation factor activity, nucleic acid binding, GO:0008135	4	1.98*E* − 5	0.008	6.3	*Tufm, Eif2ak1, Eif2s3y, *and* Eef1a2*
Transferase activity, GO:0016740	15	7.49*E* − 8	0.001	24.0	*Pdk2, St3gal3, Acvr2b, Eif2ak1, Alpk3, Pygm, Cox10, Csnk1e, Dyrk1b, Camk2b, Gamt, Prkaca, Pfkm, Ndufa10, *and* Mark2*
Regulation of system process, GO:0044057	4	1.61*E* − 4	0.04	6.3	*Ryr1, Gaa, Prkaca,* and* Casq1*
Regulation of multicellular organismal process, GO:0051239	7	1.65*E* − 4	0.04	11.0	*Ryr1, Gaa, Gnas, Gamt, Prkaca, Csda, *and* Casq1*
Regulation of metabolic process, GO:0019222	17	7.73*E* − 7	0.002	27.0	*E2f2, Camta2, Med25, Csda, Ube2l3, Cited4, Capzb, Acvr2b, Hoxc9, Eif2ak1, Trim54, Mapt, Tsc2, Spnb1, Mier2, Gnas, *and* Jmjd3*
Regulation of biological quality, GO:0065008	8	1.88*E* − 4	0.046	13.0	*Ank1, ACVR2B, TSC2, SPNB1, RYR1, GAA, PFKM, *and* CAPZB*
Porphyrin metabolic process, GO:0006778	4	1.22*E* − 7	0.001	6.3	*Ank1, EIF2AK1, COX10, *and* SPNB1*
Phosphotransferase activity, alcohol group as acceptor, GO:0016773	11	1.42*E* − 8	0.001	17.0	*Pdk2, Acvr2b, Eif2ak1, Alpk3, Csnk1e, Dyrk1b, Camk2b, Prkaca, Pfkm, Ndufa10,* and* Mark2*
Phosphate metabolic process, GO:0006796	11	1.01*E* − 7	0.001	17.0	*Pdk2, Acvr2b, Eif2ak1, Alpk3, Csnk1e, Dyrk1b, Dusp23, Atp6v0a1, Camk2b, Prkaca, *and* Mark2*
Organ development, GO:0048513	12	1.0*E* − 5	0.007	19.0	*Hsp90ab1, Ank1, Acvr2b, Hoxc9, Alpk3, Dyrk1b, Tsc2, Gaa, Gnas, Gamt, Prkaca, *and* Csda*
Nucleotide binding, GO:0000166	22	1.08*E* − 12	0.001	35.0	*Hsp90ab1, Tufm, Pdk2, Adssl1, Eif2s3y, Alpk3, Eef1a2, Pfkm, Ndufa10, Ube2l3, Idh3a, Mark2, Acvr2b, Eif2ak1, Pygm, Csnk1e, Dyrk1b, A2bp1, Gnas, Camk2b, Prkaca, *and* Myo18a*
Nitrogen compound metabolic process, GO:0006807	16	3.13*E* − 5	0.012	25.0	*E2f2, Camta2, Adssl1, Cox10, Med25, Csda, Ndufa10, Cited4, Ank1, Eif2ak1, Hoxc9, A2bp1, Spnb1, Mier2, Atp6v0a1,* and* Gamt*
Negative regulation of protein complex disassembly, GO:0043242	4	2.58*E* − 7	0.001	6.3	*Trim54, Mapt, Spnb1,* and* Capzb*
Negative regulation of biological process, GO:0048519	10	2.38*E* − 5	0.009	16.0	*Eif2ak1, Trim54, Mapt, Eef1a2, Tsc2, Spnb1, Ryr1, Jmjd3, Csda, *and* Capzb*
Muscle system process, GO:0003012	4	3.79*E* − 6	0.005	6.3	*Camta2, Ryr1, Gaa, *and* Actn3*
Heterocycle metabolic process, GO:0046483	6	5.44*E* − 6	0.006	9.5	*Ank1, ADSSL1, EIF2AK1, COX10, SPNB1, *and* ATP6V0A1*
Generation of precursor metabolites and energy, GO:0006091	8	1.13*E* − 8	0.001	13.0	*Pygm, Cox10, Gaa, Atp6v0a1, Gnas, Pfkm, Ndufa10, *and* Idh3a*
Cytoskeletal protein binding, GO:0008092	6	3.77*E* − 5	0.014	9.5	*Trim54, Spnb1, Actn3, Palld, Plec1, *and* Capzb*
Cellular protein metabolic process, GO:0044267	21	2.52*E* − 12	0.001	33.0	*Hsp90ab1, Tufm, Pdk2, Eif2s3y, Alpk3, Cox10, Eef1a2, Dusp23, Ube2l3, Mark2, Acvr2b, Rnf123, St3gal3, Eif2ak1, Fbxw5, Csnk1e, Dyrk1b, Fbxo31, Camk2b, Jmjd3, *and* Prkaca*
Cellular macromolecule catabolic process, GO:0044265	7	7.08*E* − 5	0.025	11.0	*Rnf123, Pygm, Fbxw5, Gaa, Fbxo31, Pfkm, *and* Ube2l3*
Cellular biosynthetic process, GO:0044249	17	4.59*E* − 6	0.005	27.0	*Tufm, E2f2, Camta2, Adssl1, Eif2s3y, Cox10, Eef1a2, Med25, Csda, Cited4, St3gal3, Ank1, Hoxc9, Spnb1, Mier2, Atp6v0a1,* and* Gamt*
Cation transport, GO:0006812	6	1.69*E* − 4	0.04	9.5	*Kcnc1, Ank1, Ryr1, Cacnb1, Atp6v0a1, *and* Camk2b*
Anatomical structure morphogenesis, GO:0009653	9	5.54*E* − 5	0.021	14.0	*Acvr2b, Hoxc9, Cox10, Dyrk1b, Tsc2, Gaa, Gnas, Gamt, *and* Prkaca*
GTPase activity, GO:0003924	4	4.34*E* − 5	0.015	6.3	*Tufm, Eif2s3y, Eef1a2, Gnas*

Gene set: upregulated genes					
Response to stimulus, GO:0050896	15	1.16*E* − 8	0.001	34.0	*Lyz1, Ccl21c, Ccl21a, Ccl9, Gja1, Adipoq, Ccl7, Lyzs, Cd83, Clec2d, Sepp1, Spon2, Scara5, Cd14,* and* Klf4*
Response to external stimulus, GO:0009605	7	3.16*E* − 6	0.003	16.0	*Ccl21c, Ccl21a, Ccl9, Gja1, Klf4, Cd14,* and* Ccl7*
Receptor binding, GO:0005102	8	5.33*E* − 7	0.002	18.0	*Ogn, Ccl21c, Ccl21a, Clec2d, Ccl9, Gja1, Adipoq, *and* Ccl7*
Positive regulation of biological process, GO:0048518	8	9.25*E* − 5	0.03	18.0	*Cd83, Tnfrsf12a, Ccl21a, Ccdc80, Gja1, Adipoq, Klf4, *and* Cd14*
Locomotion, GO:0040011	6	3.15*E* − 6	0.003	14.0	*Tnfrsf12a, Ccl21c, Ccl21a, Ccl9, Gja1, *and* Ccl7*
Immune response, GO:0006955	6	6.88*E* − 6	0.007	14.0	*Ccl21c, Ccl21a, Ccl9, Spon2, Cd14,* and* Ccl7*
Defense response, GO:0006952	7	5.33*E* − 7	0.002	16.0	*Lyz1, Ccl21c, Ccl21a, Clec2d, Lyzs, Cd14, *and* Ccl7*
Chemokine activity, GO:0008009	4	1.1*E* − 7	0.001	9.1	*Ccl21c, Ccl21a, Ccl9,* and* Ccl7*

**Table 2:** Enriched KEGG pathways in down- and upregulated genes (fold change > 1.7; *p* < 0.05).Only categories significantly associated (*p* value < 0.05) are considered. Kyoto Encyclopedia of Genes and Genomes (KEGG) pathways, number of enriched genes, *p* value, enrichment factor, and list of the genes enriched in each pathway are reported.

KEGG pathway	Number of genes	*p* value	Enrichment factor	Gene list
Gene set: downregulated genes				
Tight junction	2	0.0261	8.0	*Actn3, Csda*
Melanogenesis	3	8.18*E* − 4	16.5	*Camk2b, Gnas, *and* Prkaca*
Long-term depression	2	0.00871	14.3	*Ryr1, Gnas*
Arrhythmogenic right ventricular cardiomyopathy (ARVC)	2	0.00789	15.0	*Cacnb1, Actn3*
GnRH signaling pathway	3	7.52*E* − 4	17.0	*Camk2b, Gnas, *and* Prkaca*
Prostate cancer	2	0.0119	12.2	*Hsp90ab1, E2f2*
Gap junction	2	0.0119	12.2	*Gnas, Prkaca*
Galactose metabolism	2	0.00103	42.4	*Gaa, Pfkm*
Glioma	2	0.00692	16.1	*E2f2, Camk2b*
Dilated cardiomyopathy	3	5.91*E* − 4	18.4	*Cacnb1, Gnas, *and* Prkaca*
Metabolic pathways	9	2.11*E* − 4	4.34	*Adssl1, St3gal3, Cox10, Gaa, Atp6v0a1, Gamt, Pfkm, Ndufa10,* and* Idh3a*
Oxidative phosphorylation	3	0.00266	10.9	*Cox10, Atp6v0a1, *and* Ndufa10*
Calcium signaling pathway	4	3.64*E* − 4	11.9	*Ryr1, Camk2b, Gnas,* and* Prkaca*
Insulin signaling pathway	3	0.00196	12.2	*Pygm, Tsc2, *and* Prkaca*
Lysosome	2	0.0185	9.61	*Gaa, Atp6v0a1*
Taste transduction	2	0.00499	19.1	*Gnas, Prkaca*
Progesterone-mediated oocyte maturation	2	0.0157	10.5	*Hsp90ab1, Prkaca*
Parkinson's disease	2	0.0343	6.89	*Ube2l3, Ndufa10*
Long-term potentiation	2	0.00809	14.9	*Camk2b, Prkaca*
Hedgehog signaling pathway	2	0.00421	20.8	*Csnk1e, Prkaca*
Wnt signaling pathway	3	0.00276	10.8	*Csnk1e, Camk2b, *and* Prkaca*
Vascular smooth muscle contraction	2	0.0212	8.94	*Gnas, Prkaca*
MAPK signaling pathway	3	0.0132	6.11	*Mapt, Cacnb1, *and* Prkaca*
Starch and sucrose metabolism	2	0.00284	25.4	*Pygm, Gaa*

Gene set: upregulated genes				
Chemokine signaling pathway	4	1.09*E* − 4	16.2	*Ccl21c, Ccl21a, Ccl9, *and* Ccl7*
Cytokine-cytokine receptor interaction	5	1.43*E* − 5	16.2	*Tnfrsf12a, Ccl21c, Ccl21a, Ccl9, *and* Ccl7*
