# Supplementary material for: A New Transgenic Mouse Model of Heart Failure and Cardiac Cachexia Raised by Sustained Activation of Met Tyrosine Kinase in the Heart
Source: Biomed Res Int. 2016 May 19;2016:9549036. doi: 10.1155/2016/9549036 (PMC4889800; doi:10.1155/2016/9549036)
Supplement: Supplementary file 1 — Gene expression profiling was performed on GSN muscle samples from cachectic Tpr-Met mice and littermate controls. A cut-off of p<0.05 and fold change > 1.7 was used, and 107 differentially expressed genes were identified. Among these, 63 were downregulated and 44 genes were upregulated. The complete gene list is reported in Supplementary Tables 1 and 2. [file 9549036.f1.pdf]

**Supplementary Table 1: List of down-regulated transcripts (Fold Change>1.7 and p<0.05).** List of the transcipts downregulated by Tpr-Met in P27 cachectic gastrocnemius muscles. Shown are the transcripts gene annotation: *Mus Musculus* Entrez Gene ID, official symbol from NCBI, average (AVG) Log2 Ratio derived from Illumina microarrays (3 replicates/condition), fold change and p-value computations, Log2 ratio over AVG Log2 controls and full definition of the genes. All genes modulated with a fold change > 1.7 and p-value<0.05 are included.

| Entrez Gene ID | Symbol | AVG Log2 Ratio | Fold Change | T-test | Ctrl | Ctrl | Ctrl | TM | TM | TM | Definition |
|---|---|---|---|---|---|---|---|---|---|---|---|
| | | | | | | | | | | | |
| 26908 | *Eif2s3y* | -3.598 | 12.1 | 0.000 | 0.146 | -0.248 | 0.102 | -3.712 | -3.571 | -3.510 | eukaryotic translation initiation factor 2, subunit 3, structural gene Y-linked (Eif2s3y), mRNA. |
| 13628 | *Eef1a2* | -1.333 | 2.5 | 0.011 | -0.444 | 0.261 | 0.183 | -1.503 | -0.950 | -1.546 | eukaryotic translation elongation factor 1 alpha 2 (Eef1a2), mRNA. |
| 13628 | *Eef1a2* | -1.298 | 2.5 | 0.031 | -0.383 | 0.275 | 0.108 | -1.632 | -0.605 | -1.656 | eukaryotic translation elongation factor 1 alpha 2 (Eef1a2), mRNA. |
| 56222 | *Cited4* | -1.268 | 2.4 | 0.029 | -0.466 | 0.035 | 0.431 | -1.109 | -0.889 | -1.805 | Cbp/p300-interacting transactivator, with Glu/Asp-rich carboxy-terminal domain, 4 (Cited4), mRNA. |
| 18810 | *Plec1* | -1.246 | 2.4 | 0.022 | -0.440 | 0.327 | 0.113 | -1.303 | -0.779 | -1.657 | plectin 1 (Plec1), transcript variant 10, mRNA. |
| 20741 | *Spnb1* | -1.238 | 2.4 | 0.016 | -0.384 | 0.314 | 0.071 | -1.129 | -0.902 | -1.684 | spectrin beta 1 (Spnb1), mRNA. |
| 18642 | *Pfkm* | -1.190 | 2.3 | 0.016 | -0.280 | -0.031 | 0.311 | -1.655 | -0.829 | -1.085 | phosphofructokinase, muscle (Pfkm), mRNA. |
| 100046056 | *LOC100046056* | -1.143 | 2.2 | 0.039 | -0.275 | 0.533 | -0.258 | -1.516 | -0.620 | -1.294 | PREDICTED: similar to Pre-B-cell leukemia transcription factor interacting protein 1 (LOC100046056), mRNA. |
| 13628 | *Eef1a2* | -1.108 | 2.2 | 0.036 | -0.295 | 0.250 | 0.045 | -1.431 | -0.472 | -1.420 | eukaryotic translation elongation factor 1 alpha 2 (Eef1a2), mRNA. |
| 12323 | *Camk2b* | -1.089 | 2.1 | 0.015 | -0.332 | 0.319 | 0.013 | -1.163 | -0.728 | -1.376 | calcium/calmodulin-dependent protein kinase II, beta (Camk2b), mRNA. |
| 20441 | *St3gal3* | -1.076 | 2.1 | 0.010 | -0.377 | 0.337 | 0.039 | -1.166 | -0.864 | -1.199 | ST3 beta-galactoside alpha-2,3-sialyltransferase 3 (St3gal3), mRNA. |
| 20190 | *Ryr1* | -1.073 | 2.1 | 0.033 | -0.468 | 0.502 | -0.034 | -0.945 | -0.838 | -1.435 | ryanodine receptor 1, skeletal muscle (Ryr1), mRNA. |
| 19309 | *Pygm* | -1.070 | 2.1 | 0.025 | -0.325 | 0.319 | 0.006 | -1.289 | -0.581 | -1.341 | muscle glycogen phosphorylase (Pygm), mRNA. |
| 268859 | *A2bp1* | -1.069 | 2.1 | 0.013 | -0.059 | -0.139 | 0.198 | -1.432 | -0.649 | -1.125 | RNA binding protein, fox-1 homolog (C. elegans) 1, mRNA |
| 216874 | *Camta2* | -1.045 | 2.1 | 0.029 | -0.370 | 0.219 | 0.151 | -1.314 | -0.542 | -1.281 | calmodulin binding transcription activator 2 (Camta2), mRNA. |
| 11733 | *Ank1* | -1.039 | 2.1 | 0.028 | -0.407 | 0.155 | 0.253 | -1.196 | -0.590 | -1.330 | ankyrin 1, erythroid (Ank1), mRNA. |
| 68440 | *Dusp23* | -1.019 | 2.0 | 0.032 | -0.366 | 0.119 | 0.247 | -1.483 | -0.600 | -0.974 | dual specificity phosphatase 23 (Dusp23), mRNA. |
| 14683 | *Gnas* | -1.013 | 2.0 | 0.044 | -0.464 | 0.105 | 0.360 | -1.495 | -0.652 | -0.893 | GNAS (guanine nucleotide binding protein, alpha stimulating) complex locus (Gnas), transcript variant 3, mRNA. |
| 12372 | *Casq1* | -1.013 | 2.0 | 0.000 | 0.016 | -0.006 | -0.010 | -1.167 | -0.912 | -0.962 | calsequestrin 1 (Casq1), nuclear gene encoding mitochondrial protein, mRNA. |

| | | | | | | | | | | | |
|---|---|---|---|---|---|---|---|---|---|---|---|
| 56449 | *Csda* | -1.013 | 2.0 | 0.026 | -0.433 | 0.054 | 0.379 | -1.355 | -0.797 | -0.887 | cold shock domain protein A (Csda), mRNA. |
| 233335 | *Synm* | -0.994 | 2.0 | 0.005 | -0.330 | 0.281 | 0.049 | -1.014 | -0.966 | -1.001 | synemin, intermediate filament protein (Synm), transcript variant 3, mRNA. |
| 76454 | *Fbxo31* | -0.992 | 2.0 | 0.026 | -0.303 | 0.380 | -0.078 | -1.310 | -0.610 | -1.055 | F-box protein 31 (Fbxo31), mRNA. |
| 72333 | *Palld* | -0.992 | 2.0 | 0.020 | -0.264 | 0.362 | -0.098 | -1.239 | -0.620 | -1.116 | palladin, cytoskeletal associated protein (Palld), mRNA. |
| 14683 | *Gnas* | -0.990 | 2.0 | 0.032 | -0.386 | 0.000 | 0.386 | -1.411 | -0.741 | -0.818 | GNAS (guanine nucleotide binding protein, alpha stimulating) complex locus (Gnas), transcript variant 8, mRNA. |
| 15516 | *Hsp90ab1* | -0.989 | 2.0 | 0.013 | -0.232 | 0.067 | 0.165 | -1.163 | -0.587 | -1.217 | heat shock protein 90kDa alpha (cytosolic), class B member 1 (Hsp90ab1), mRNA. |
| 360013 | *Myo18a* | -0.983 | 2.0 | 0.020 | -0.349 | 0.363 | -0.015 | -1.013 | -0.691 | -1.244 | myosin XVIIIa (Myo18a), mRNA. |
| 12295 | *Cacnb1* | -0.972 | 2.0 | 0.030 | -0.402 | 0.398 | 0.005 | -1.042 | -0.624 | -1.250 | calcium channel, voltage-dependent, beta 1 subunit (Cacnb1), mRNA |
| 75613 | *Med25* | -0.970 | 2.0 | 0.023 | -0.349 | 0.329 | 0.020 | -1.183 | -0.593 | -1.133 | mediator of RNA polymerase II transcription, subunit 25 homolog (yeast) (Med25), mRNA. |
| 27373 | *Csnk1e* | -0.962 | 1.9 | 0.016 | -0.345 | 0.212 | 0.134 | -1.259 | -0.701 | -0.926 | casein kinase 1, epsilon (Csnk1e), mRNA. |
| 14431 | *Gamt* | -0.954 | 1.9 | 0.038 | -0.186 | 0.171 | 0.015 | -0.859 | -0.495 | -1.506 | guanidinoacetate methyltransferase (Gamt), mRNA. |
| 15516 | *Hsp90ab1* | -0.949 | 1.9 | 0.045 | -0.381 | 0.366 | 0.015 | -1.262 | -0.457 | -1.126 | heat shock protein 90kDa alpha (cytosolic), class B member 1 (Hsp90ab1), mRNA. |
| 11481 | *Acvr2b* | -0.946 | 1.9 | 0.004 | -0.237 | 0.178 | 0.060 | -0.806 | -0.906 | -1.126 | activin receptor IIB (Acvr2b), mRNA. |
| 13728 | *Mark2* | -0.945 | 1.9 | 0.027 | -0.238 | 0.263 | -0.025 | -1.350 | -0.527 | -0.959 | MAP/microtubule affinity-regulating kinase 2 (Mark2), transcript variant 4, mRNA. |
| 11733 | *Ank1* | -0.916 | 1.9 | 0.043 | -0.472 | 0.145 | 0.327 | -0.977 | -0.546 | -1.225 | ankyrin 1, erythroid (Ank1), mRNA. |
| 13549 | *Dyrk1b* | -0.915 | 1.9 | 0.028 | -0.391 | 0.269 | 0.122 | -1.147 | -0.550 | -1.049 | dual-specificity tyrosine-(Y)-phosphorylation regulated kinase 1b (Dyrk1b), transcript variant 1, mRNA. |
| 58522 | *Trim54* | -0.906 | 1.9 | 0.045 | -0.328 | 0.196 | 0.132 | -1.092 | -0.381 | -1.247 | tripartite motif-containing 54 (Trim54), mRNA. |
| 17762 | *Mapt* | -0.906 | 1.9 | 0.039 | -0.265 | 0.338 | -0.073 | -1.009 | -0.449 | -1.261 | microtubule-associated protein tau (Mapt), mRNA |
| 67834 | *Idh3a* | -0.904 | 1.9 | 0.031 | -0.190 | 0.231 | -0.041 | -0.861 | -0.495 | -1.357 | isocitrate dehydrogenase 3 (NAD+) alpha (Idh3a), nuclear gene encoding mitochondrial protein, mRNA. |
| 14387 | *Gaa* | -0.895 | 1.9 | 0.011 | -0.269 | 0.184 | 0.084 | -0.952 | -0.617 | -1.115 | glucosidase, alpha, acid (Gaa), mRNA. |
| 11975 | *Atp6v0a1* | -0.894 | 1.9 | 0.047 | -0.387 | 0.285 | 0.101 | -1.201 | -0.415 | -1.067 | ATPase, H+ transporting, lysosomal V0 subunit A1 (Atp6v0a1), mRNA |
| 68097 | *Dynll2* | -0.892 | 1.9 | 0.012 | -0.252 | 0.098 | 0.154 | -1.059 | -0.570 | -1.048 | dynein light chain LC8-type 2 (Dynll2), mRNA. |
| 18747 | *Prkaca* | -0.891 | 1.9 | 0.018 | -0.260 | 0.093 | 0.167 | -1.009 | -0.525 | -1.140 | protein kinase, cAMP dependent, catalytic, alpha (Prkaca), mRNA. |
| 239102 | *Zfhx2* | -0.890 | 1.9 | 0.026 | -0.229 | 0.275 | -0.046 | -1.074 | -0.468 | -1.128 | zinc finger homeobox 2 (Zfhx2), mRNA. |
| 70209 | *Tmem143* | -0.888 | 1.9 | 0.048 | -0.521 | 0.365 | 0.156 | -1.087 | -0.556 | -1.020 | transmembrane protein 143 (Tmem143), mRNA. |

| | | | | | | | | | | | |
|---|---|---|---|---|---|---|---|---|---|---|---|
| 20340 | *Glg1* | -0.884 | 1.8 | 0.024 | -0.157 | 0.366 | -0.210 | -0.911 | -0.579 | -1.162 | golgi apparatus protein 1 (Glg1), mRNA. |
| 11906 | *Zfhx3* | -0.883 | 1.8 | 0.033 | -0.463 | 0.166 | 0.297 | -1.170 | -0.678 | -0.801 | zinc finger homeobox 3 (Zfhx3), mRNA. |
| 15516 | *Hsp90ab1* | -0.876 | 1.8 | 0.018 | -0.377 | 0.164 | 0.213 | -1.039 | -0.637 | -0.953 | heat shock protein 90kDa alpha (cytosolic), class B member 1 (Hsp90ab1), mRNA. |
| 22195 | *Ube2l3* | -0.870 | 1.8 | 0.027 | -0.301 | 0.217 | 0.084 | -1.001 | -0.472 | -1.138 | ubiquitin-conjugating enzyme E2L 3 (Ube2l3), mRNA. |
| 22084 | *Tsc2* | -0.857 | 1.8 | 0.026 | -0.299 | 0.491 | -0.192 | -0.857 | -0.843 | -0.869 | Mtuberous sclerosis 2 (Tsc2), transcript variant 2, mRNA. |
| 242705 | *E2f2* | -0.855 | 1.8 | 0.024 | 0.040 | 0.243 | -0.283 | -0.636 | -0.699 | -1.229 | E2F transcription factor 2 (E2f2), mRNA. |
| 67273 | *Ndufa10* | -0.850 | 1.8 | 0.034 | -0.244 | 0.205 | 0.039 | -1.056 | -0.381 | -1.114 | NADH dehydrogenase (ubiquinone) 1 alpha subcomplex 10 (Ndufa10), nuclear gene encoding mitochondrial protein, mRNA. |
| 11565 | *Adssl1* | -0.849 | 1.8 | 0.050 | -0.305 | -0.054 | 0.359 | -1.317 | -0.555 | -0.676 | adenylosuccinate synthetase like 1 (Adssl1), mRNA. |
| 116904 | *Alpk3* | -0.845 | 1.8 | 0.015 | -0.207 | 0.306 | -0.099 | -1.104 | -0.638 | -0.794 | alpha-kinase 3 (Alpk3), mRNA. |
| 22195 | *Ube2l3* | -0.843 | 1.8 | 0.015 | -0.259 | 0.148 | 0.111 | -0.941 | -0.534 | -1.054 | ubiquitin-conjugating enzyme E2L 3 (Ube2l3), mRNA. |
| 239102 | *Zfhx2* | -0.843 | 1.8 | 0.013 | -0.173 | 0.123 | 0.050 | -1.125 | -0.519 | -0.886 | zinc finger homeobox 2 (Zfhx2), mRNA. |
| 230721 | *Pabpc4* | -0.839 | 1.8 | 0.046 | -0.467 | 0.260 | 0.206 | -1.033 | -0.487 | -0.996 | poly A binding protein, cytoplasmic 4 (Pabpc4), transcript variant 2, mRNA. |
| 84585 | *Rnf123* | -0.835 | 1.8 | 0.037 | -0.459 | 0.306 | 0.153 | -0.976 | -0.555 | -0.975 | ring finger protein 123 (Rnf123), mRNA. |
| 22084 | *Tsc2* | -0.827 | 1.8 | 0.012 | -0.258 | 0.287 | -0.028 | -0.769 | -0.681 | -1.032 | tuberous sclerosis 2 (Tsc2), transcript variant 1, mRNA. |
| 70383 | *Cox10* | -0.825 | 1.8 | 0.036 | -0.255 | 0.097 | 0.158 | -1.122 | -0.369 | -0.985 | COX10 homolog, cytochrome c oxidase assembly protein, heme A: farnesyltransferase (yeast) (Cox10), nuclear gene encoding mitochondrial protein, mRNA. |
| 224727 | *Bat3* | -0.825 | 1.8 | 0.041 | -0.405 | 0.252 | 0.153 | -1.015 | -0.450 | -1.009 | HLA-B-associated transcript 3 (Bat3), mRNA. |
| 15467 | *Eif2ak1* | -0.817 | 1.8 | 0.012 | -0.076 | 0.197 | -0.121 | -0.911 | -0.508 | -1.030 | eukaryotic translation initiation factor 2 alpha kinase 1 (Eif2ak1), mRNA |
| 110198 | *Akr7a5* | -0.812 | 1.8 | 0.040 | -0.443 | 0.282 | 0.161 | -1.096 | -0.572 | -0.768 | aldo-keto reductase family 7, member A5 (aflatoxin aldehyde reductase) (Akr7a5), mRNA. |
| 18604 | *Pdk2* | -0.811 | 1.8 | 0.033 | -0.296 | 0.310 | -0.014 | -1.118 | -0.479 | -0.836 | pyruvate dehydrogenase kinase, isoenzyme 2 (Pdk2), mRNA. |
| 22378 | *Wbp2* | -0.810 | 1.8 | 0.033 | -0.238 | 0.167 | 0.070 | -1.089 | -0.371 | -0.968 | WW domain binding protein 2 (Wbp2), mRNA. |
| 216850 | *Jmjd3* | -0.796 | 1.7 | 0.032 | -0.426 | 0.102 | 0.324 | -0.682 | -0.699 | -1.006 | jumonji domain containing 3 (Jmjd3), mRNA. |
| 11474 | *Actn3* | -0.791 | 1.7 | 0.037 | -0.410 | 0.368 | 0.042 | -0.573 | -0.794 | -1.006 | actinin alpha 3 (Actn3), mRNA. |
| 16502 | *Kcnc1* | -0.791 | 1.7 | 0.004 | -0.116 | 0.113 | 0.003 | -1.021 | -0.707 | -0.644 | potassium voltage gated channel, Shaw-related subfamily, member 1 (Kcnc1), mRNA. |
| 69585 | *Hfe2* | -0.782 | 1.7 | 0.017 | -0.365 | 0.077 | 0.288 | -0.827 | -0.674 | -0.846 | hemochromatosis type 2 (juvenile) (human homolog) (Hfe2), mRNA. |
| 76454 | *Fbxo31* | -0.780 | 1.7 | 0.022 | -0.321 | 0.252 | 0.069 | -0.930 | -0.520 | -0.889 | F-box protein 31 (Fbxo31), mRNA. |

| | | | | | | | | | | | |
|---|---|---|---|---|---|---|---|---|---|---|---|
| 12345 | *Capzb* | -0.776 | 1.7 | 0.031 | -0.324 | 0.151 | 0.173 | -0.872 | -0.439 | -1.018 | capping protein (actin filament) muscle Z-line, beta (Capzb), transcript variant 1, mRNA. |
| 233870 | *Tufm* | -0.774 | 1.7 | 0.036 | -0.318 | 0.265 | 0.053 | -0.819 | -0.441 | -1.063 | Tu translation elongation factor, mitochondrial (Tufm), nuclear gene encoding mitochondrial protein, mRNA. |
| 30839 | *Fbxw5* | -0.774 | 1.7 | 0.024 | -0.296 | 0.173 | 0.123 | -0.874 | -0.459 | -0.988 | F-box and WD-40 domain protein 5 (Fbxw5), mRNA. |
| 15427 | *Hoxc9* | -0.769 | 1.7 | 0.041 | -0.399 | 0.145 | 0.254 | -0.926 | -0.444 | -0.935 | homeo box C9 (Hoxc9), mRNA. |
| 70427 | *Mier2* | -0.766 | 1.7 | 0.021 | -0.245 | 0.158 | 0.087 | -0.958 | -0.438 | -0.902 | mesoderm induction early response 1, family member 2 (Mier2), mRNA |

**Supplementary Table 2: List of up-regulated genes (Fold Change>1.7 and p<0.05).** List of the transcipts upregulated by Tpr-Met in P27 cachectic gastrocnemius muscles. Shown are the transcripts gene annotation: *Mus Musculus* Entrez Gene ID, official symbol from NCBI, average (AVG) Log2 Ratio derived from Illumina microarrays (3 replicates/condition), fold change and p-value computations, Log2 ratio over AVG Log2 control values and full definition of the genes. All genes modulated with a fold change > 1.7 and p-value<0.05 are included.

| Entrez Gene ID | Symbol | AVG Log2 Ratio | Fold Change | T-test | Ctrl | Ctrl | Ctrl | TM | TM | TM | Definition |
|---|---|---|---|---|---|---|---|---|---|---|---|
| | | | | | | | | | | | |
| 21754 | *Tesk1* | 2.003 | 4.0 | 0.000 | -0.266 | 0.157 | 0.109 | 1.965 | 2.166 | 1.879 | testis specific protein kinase 1 (Tesk1), mRNA. |
| 20306 | *Ccl7* | 1.617 | 3.1 | 0.047 | 0.234 | -0.006 | -0.228 | 0.621 | 1.689 | 2.541 | chemokine (C-C motif) ligand 7 (Ccl7), mRNA. |
| 17841 | *Mup2* | 1.420 | 2.7 | 0.004 | 0.263 | 0.037 | -0.300 | 1.207 | 1.760 | 1.292 | major urinary protein 2 (Mup2), transcript variant 1, mRNA. |
| 107652 | *Uap1* | 1.380 | 2.6 | 0.034 | 0.321 | -0.289 | -0.032 | 0.581 | 1.725 | 1.833 | UDP-N-acetylglucosamine pyrophosphorylase 1 (Uap1), mRNA. |
| 20308 | *Ccl9* | 1.328 | 2.5 | 0.041 | 0.347 | -0.288 | -0.059 | 0.566 | 1.460 | 1.957 | chemokine (C-C motif) ligand 9 (Ccl9), mRNA. |
| 15439 | *Hp* | 1.209 | 2.3 | 0.013 | -0.039 | 0.301 | -0.262 | 0.792 | 1.577 | 1.259 | haptoglobin (Hp), mRNA. |
| 12522 | *Cd83* | 1.180 | 2.3 | 0.014 | 0.383 | -0.082 | -0.302 | 0.889 | 1.097 | 1.553 | CD83 antigen (Cd83), mRNA. |
| 20229 | *LOC100047261* | 1.133 | 2.2 | 0.013 | 0.337 | -0.389 | 0.052 | 1.195 | 0.829 | 1.376 | PREDICTED:similar to spermidine/spermine N1-acetyltransferase (LOC100047261), misc RNA. |
| 20194 | *S100a10* | 1.123 | 2.2 | 0.039 | 0.264 | -0.515 | 0.251 | 0.681 | 1.090 | 1.599 | S100 calcium binding protein A10 (calpactin) (S100a10), mRNA. |
| 22320 | *Vamp8* | 1.106 | 2.2 | 0.050 | 0.298 | 0.416 | -0.714 | 0.863 | 1.017 | 1.437 | vesicle-associated membrane protein 8 (Vamp8), mRNA. |
| 13733 | *Emr1* | 1.073 | 2.1 | 0.009 | 0.218 | -0.283 | 0.065 | 0.930 | 0.884 | 1.405 | EGF-like module containing, mucin-like, hormone receptor-like sequence 1 (Emr1), mRNA. |
| 13106 | *Cyp2e1* | 1.065 | 2.1 | 0.007 | -0.165 | 0.387 | -0.221 | 0.962 | 1.192 | 1.040 | cytochrome P450, family 2, subfamily e, polypeptide 1 (Cyp2e1), mRNA. |
| 11450 | *Adipoq* | 1.061 | 2.1 | 0.026 | 0.340 | -0.034 | -0.306 | 0.696 | 0.965 | 1.520 | adiponectin, C1Q and collagen domain containing (Adipoq), mRNA. |
| 17105 | *Lyzs* | 1.054 | 2.1 | 0.030 | 0.441 | -0.512 | 0.071 | 0.741 | 1.157 | 1.263 | lysozyme (Lyzs), mRNA. |
| 232087 | *Mat2a* | 1.033 | 2.0 | 0.013 | 0.178 | 0.244 | -0.422 | 1.263 | 0.909 | 0.927 | methionine adenosyltransferase II, alpha (Mat2a), mRNA. |
| 12825 | *Col3a1* | 1.004 | 2.0 | 0.009 | 0.092 | -0.386 | 0.294 | 0.883 | 1.025 | 1.104 | collagen, type III, alpha 1 (Col3a1), mRNA. |
| 100689 | *Spon2* | 0.996 | 2.0 | 0.034 | 0.189 | -0.001 | -0.187 | 1.457 | 1.086 | 0.444 | spondin 2, extracellular matrix protein (Spon2), mRNA. |
| 12475 | *Cd14* | 0.970 | 2.0 | 0.026 | 0.211 | -0.400 | 0.189 | 0.572 | 1.161 | 1.178 | CD14 antigen (Cd14), mRNA. |
| 620807 | *OTTMUSG000* | 0.968 | 2.0 | 0.012 | 0.193 | 0.022 | -0.215 | 0.608 | 1.254 | 1.042 | predicted gene, 620807 (620807), mRNA. |
| 16600 | *Klf4* | 0.957 | 1.9 | 0.021 | -0.132 | -0.195 | 0.327 | 1.355 | 0.766 | 0.749 | Kruppel-like factor 4 (gut) (Klf4), mRNA. |
| 13479 | *Dpep1* | 0.948 | 1.9 | 0.025 | 0.111 | -0.317 | 0.206 | 0.513 | 1.156 | 1.175 | dipeptidase 1 (renal) (Dpep1), mRNA. |
| 18295 | *Ogn* | 0.940 | 1.9 | 0.045 | 0.348 | -0.522 | 0.174 | 0.625 | 0.916 | 1.278 | osteoglycin (Ogn), mRNA. |
| 65956 | *Ccl21c* | 0.934 | 1.9 | 0.000 | 0.146 | -0.075 | -0.071 | 0.902 | 0.948 | 0.952 | chemokine (C-C motif) ligand 21c (leucine) (Ccl21c), mRNA. |
| 18295 | *Ogn* | 0.932 | 1.9 | 0.029 | 0.162 | -0.360 | 0.198 | 0.720 | 0.721 | 1.355 | osteoglycin (Ogn), mRNA. |
| 27279 | *Tnfrsf12a* | 0.929 | 1.9 | 0.048 | 0.064 | -0.413 | 0.349 | 0.535 | 0.874 | 1.377 | tumor necrosis factor receptor superfamily, member 12a (Tnfrsf12a), mRNA. |
| 70717 | *6330406I15Rik* | 0.928 | 1.9 | 0.044 | 0.338 | -0.587 | 0.249 | 0.724 | 0.906 | 1.153 | RIKEN cDNA 6330406I15 gene (6330406I15Rik), mRNA. |
| 11745 | *Anxa3* | 0.926 | 1.9 | 0.039 | 0.564 | -0.458 | -0.105 | 0.931 | 0.811 | 1.035 | annexin A3 (Anxa3), mRNA. |

| | | | | | | | | | | | |
|---|---|---|---|---|---|---|---|---|---|---|---|
| 104725 | *1110002B05Rik* | 0.912 | 1.9 | 0.010 | 0.063 | -0.090 | 0.028 | 0.814 | 0.642 | 1.280 | RIKEN cDNA 1110002B05 gene (1110002B05Rik), mRNA. |
| 13733 | *Emr1* | 0.890 | 1.9 | 0.023 | 0.250 | -0.415 | 0.166 | 0.646 | 0.906 | 1.117 | EGF-like module containing, mucin-like, hormone receptor-like sequence 1 (Emr1), mRNA. |
| 17110 | *Lyz1* | 0.884 | 1.8 | 0.037 | 0.072 | 0.090 | -0.163 | 0.402 | 0.897 | 1.353 | lysozyme 1 (Lyz1), mRNA. |
| 71145 | *Scara5* | 0.879 | 1.8 | 0.026 | 0.283 | -0.472 | 0.190 | 0.744 | 0.834 | 1.058 | scavenger receptor class A, member 5 (putative) (Scara5), mRNA. |
| 64661 | *Krtdap* | 0.860 | 1.8 | 0.040 | 0.234 | -0.261 | 0.027 | 0.464 | 0.798 | 1.318 | keratinocyte differentiation associated protein (Krtdap), mRNA. XM_923930 XM_923934 |
| 18829 | *Ccl21a* | 0.851 | 1.8 | 0.000 | 0.127 | -0.007 | -0.120 | 0.876 | 0.906 | 0.770 | chemokine (C-C motif) ligand 21A (Ccl21a), mRNA. |
| 78889 | *Wsb1* | 0.845 | 1.8 | 0.047 | 0.466 | -0.409 | -0.056 | 1.154 | 0.713 | 0.667 | WD repeat and SOCS box-containing 1 (Wsb1), transcript variant 2, mRNA. |
| 56429 | *Dpt* | 0.842 | 1.8 | 0.028 | 0.303 | -0.429 | 0.126 | 0.630 | 0.861 | 1.036 | dermatopontin (Dpt), mRNA. |
| 80891 | *Msr2* | 0.830 | 1.8 | 0.038 | 0.285 | -0.507 | 0.222 | 0.645 | 0.895 | 0.950 | macrophage scavenger receptor 2 (Msr2), mRNA. |
| 68701 | *Dysfip1* | 0.829 | 1.8 | 0.045 | 0.386 | -0.343 | -0.043 | 0.689 | 0.584 | 1.214 | dysferlin interacting protein 1 (Dysfip1), mRNA. |
| 20363 | *Sepp1* | 0.827 | 1.8 | 0.029 | 0.249 | -0.228 | -0.021 | 0.555 | 0.697 | 1.229 | selenoprotein P, plasma, 1 (Sepp1), transcript variant 2, mRNA. |
| 67896 | *Ccdc80* | 0.823 | 1.8 | 0.017 | 0.216 | -0.237 | 0.021 | 0.673 | 1.147 | 0.647 | coiled-coil domain containing 80 (Ccdc80), mRNA. |
| 15199 | *Hebp1* | 0.816 | 1.8 | 0.025 | -0.018 | -0.308 | 0.326 | 0.597 | 0.769 | 1.083 | heme binding protein 1 (Hebp1), mRNA. |
| 215280 | *Wipf1* | 0.789 | 1.7 | 0.017 | 0.118 | -0.318 | 0.200 | 0.661 | 0.678 | 1.028 | WAS/WASL interacting protein family, member 1 (Wipf1), mRNA. |
| 110454 | *Ly6a* | 0.774 | 1.7 | 0.048 | 0.401 | -0.291 | -0.111 | 0.438 | 0.826 | 1.057 | lymphocyte antigen 6 complex, locus A (Ly6a), mRNA. |
| 19231 | *Ptma* | 0.773 | 1.7 | 0.004 | -0.018 | -0.123 | 0.141 | 0.781 | 0.589 | 0.949 | PREDICTED: similar to prothymosin alpha (LOC100044779), misc RNA. |
| 93694 | *Clec2d* | 0.773 | 1.7 | 0.030 | 0.423 | -0.280 | -0.142 | 0.689 | 0.671 | 0.958 | C-type lectin domain family 2, member d (Clec2d), mRNA. |
| 13723 | *Emb* | 0.772 | 1.7 | 0.028 | 0.281 | -0.318 | 0.037 | 0.606 | 0.642 | 1.068 | embigin (Emb), mRNA. |
| 14609 | *Gja1* | 0.767 | 1.7 | 0.024 | 0.294 | -0.395 | 0.101 | 0.883 | 0.769 | 0.650 | gap junction membrane channel protein alpha 1 (Gja1), mRNA. |
